# A Village-Based Intervention: Promoting Folic Acid Use among Rural Chinese Women

**DOI:** 10.3390/nu9020174

**Published:** 2017-02-21

**Authors:** Qian Lin, Lina Yang, Fang Li, Hong Qin, Mingzhi Li, Jihua Chen, Jing Deng, Xiangying Hu

**Affiliations:** 1 Department of Nutrition Science and Food Hygiene, Xiangya School of Public Health, Central South University, 110 Xiangya Road, Changsha 410078, Hunan, China; squall21358993@hotmail.com (F.L.); qinhong@csu.edu.cn (H.Q.); lmz1976@126.com (M.L.); chenjh@csu.edu.cn (J.C.); hxyybyq@163.com (X.H.); 2 Department of Epidemiology and Statistical Science, Xiangya School of Public Health, Central South University, 110 Xiangya Road, Changsha 410078, Hunan, China; dengjing2@126.com

**Keywords:** folic acid, neural tube defects, rural, women at birth age, village-based intervention, China

## Abstract

Background: Folic acid supplementation is effective in reducing the risk of neural tube defects (NTDs). However, the use of folic acid is low among rural women in China. Nutrition education can provide information about folic acid and encourage its use. The primary objective of this study was to test the effectiveness of a village-based nutrition intervention on folic acid use among rural women. Methods: Sixty villages were randomly selected using multiple-stage sampling and were divided into control and intervention groups. The intervention included nutritional education at village clinics, written materials, and text messages (SMS). Folic acid use knowledge and behavior was assessed at baseline and after the intervention. Results: Self-reported compliance with folic acid supplement use increased from 17.0%–29.2% at baseline to 41.7%–59.2% one year post-intervention. During the same period, the folic acid knowledge score in the intervention group increased from 3.07 to 3.65, significantly higher than the control group (3.11 to 3.35). Multivariate binary logistic regression showed that the women who received folic acid education and SMS intervention were more likely to comply with folic acid supplement recommendations. Conclusions: The results indicated that an integrated village-based folic acid education intervention may be an effective way of promoting folic acid use for the prevention of NTDs in rural women.

## 1. Introduction

Neural tube defects (NTDs) are the most common birth defects that contribute to infant mortality and serious disability [1]. NTDs occur in approximately the third or fourth week of pregnancy, before many women even know that they are pregnant. NTDs are highly prevalent in China. National birth surveillance showed that the NTD incidence is still high in rural areas of China, occurring in 12.9 of 10,000 births in 2007. The distribution of NTDs varies by region, with a higher prevalence in the north compared with the south and in rural compared to urban populations [2]. NTDs are preventable if women consume a daily supplement of 400 μg folic acid (FA) before and during conception. Therefore, many governments (including China’s) recommend that all women take a daily folic acid supplement for 6 months, starting from 3 months before conception and continuing throughout the first trimester. Some research has indicated that blood folate deficiency is high among Chinese adults [3,4]. However, there are disparities in the consumption of folic acid in China, with 24.69%–41.0% of rural women reporting daily folic acid consumption compared with 57.1%–86.58% of urban women [5,6,7,8].

Since 1993, the Chinese government has developed strategies targeting women of child-bearing age to promote the use of folic acid for the prevention of NTDs. In some western regions with a high risk of NTDs, folic acid fortification of flour or rice has been reported as a potential health strategy to tackle folate deficiency [9,10]. However, small privately-owned flour milling factories account for a large proportion in China. Flour fortified with folic acid is not easy to implement broadly, because the specialized techniques require high quality control. Moreover, a shortage of related regulations and standards hinders the development of the folic acid-fortified food in China. For the time being, China still adopts folic acid supplements to prevent neural-tube defects. One strategy that provided free folic acid supplements in women’s hospitals was interrupted with the abolition of mandatory premarital check-ups in 2003. In the meantime, the incidence of birth defects including NTDs in China has increased [11]. In June 2009, a health policy called “Supplementing Folic Acid to Prevent NTD” (SFAPN) was announced by the Chinese government. Under this policy, rural women of childbearing age can receive a daily supplement of 400 μg of folic acid supplied free at village health posts. The Chinese health authority aims for 60% of the targeted women to know about folic acid and for 60% of them to be taking folic acid by the end of 2009.

Although free folic acid supplements have been provided for rural women, lack of compliance has been a barrier to the program’s success. The level of folic acid awareness and adequate intake remain relatively low in rural regions of China—below 30% in some villages [12]. Improving folic acid knowledge is an essential step in promoting the use of folic acid to prevent NTDs [13,14]. The major barrier for the effective delivery of folic acid knowledge is limited knowledge among village health workers. A study examining folic acid awareness among primary health workers showed that only 39.0%–48.1% of the respondents knew the benefits of folic acid supplementation [15]. No previous research has been conducted on how folic acid knowledge and folic acid supplementation can be increased in villages.

The aim of this article is to evaluate the results of a village-based folic acid education intervention to increase the knowledge and use of folic acid to prevent NTDs among rural women.

## 2. Methods

### 2.1. Study Design

This study was conducted in Hunan province in central south China. A recent study showed that alongside the decline of birth defects in northern regions with a high risk of NTDs, the incidence of birth defects gradually increased in some southern regions of China [16]. In Hunan province, the incidence of birth defects increased from 10.709/1000 in 1996–2004 to 13.836/1000 in 2005–2012 [16], and was even higher in rural regions (26/1000 in 2009, the SFAPN office of Hunan).

Multi-stage sampling based on NTD incidence was used to select townships that represented the same levels of SFAPN implementation in Changsha county in Hunan province. Ten townships were selected using simple random sampling. Then, six SFAPN villages were selected from each township using simple random sampling, and the sixty total selected villages were randomly assigned to the control group or the intervention group. The two groups were matched in terms of birth rate, NTD incidence, population size, economy level, and when SFAPN started in the village. For each SFAPN village, all the women of childbearing age who planned to have a baby were selected using systematic sampling.

Based on the pilot study results, we anticipated differences of 20% in compliance with folic acid use, ranging from 30% to 50%. The sample size for an average cluster size of 10 women was calculated for 80% power and 95% of the primary outcome, resulting in approximately 300 women in each intervention and control group. An intervention was implemented in all 30 intervention villages for one year. It consisted of folic acid education at village clinics and SMS messages regarding folic acid information for rural women. The unit of randomization was the village. Entry criteria for villages were as follows: (1) a minimum of 30 women planning pregnancy in the village; (2) the presence of a village health post. Entry criteria for the women were (1) childbearing age and (2) planning a pregnancy.

### 2.2. Ethical Approval

This study was approved by the independent ethics committee of the Institute of Clinical Pharmacology, Central South University (project number CTXY-110013, December 2011). Written informed consent was obtained from all the subjects.

### 2.3. Intervention

**Figure 1:**
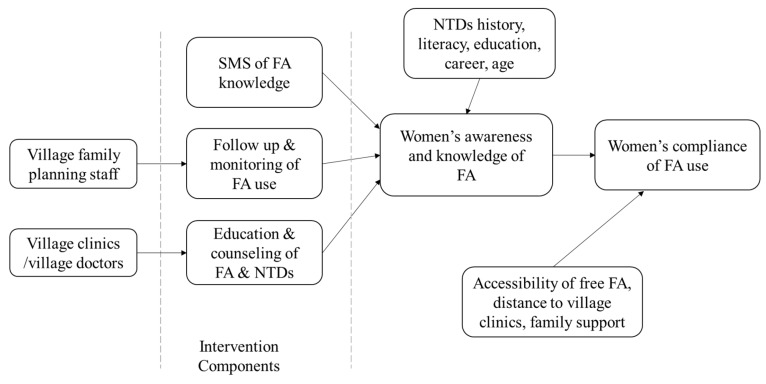
Pathway of the intervention and primary outcome. FA: folic acid; NTD: neural tube defect.

All village doctors and family planning staff in the intervention villages received a two-day training program delivered by the Changsha county health department. The duration of the folic acid intervention for the women in the intervention group was one year, from September 2013 to September 2014. Figure 1 shows the pathway of the intervention: (1) Folic acid education: folic acid education and counseling sessions were provided monthly in village clinics by village doctors; (2) Monitoring of folic acid use: family planning staff in the intervention group informed every participant of the free folic acid supplements available at the village health posts and followed up their adherence to folic acid use monthly; (3) SMS intervention: the women who consented received short SMS text messages designed to address a range of common issues with folic acid supplements, including the benefits of folic acid, where and when to obtain free folic acid supplements, and the rules for folic acid supplementation (dose, frequency, and period). Five SMS text messages were delivered automatically twice per month via an open source integrated communication service called Fetion. It cost RMB 0.6 (USD 0.1) per month per woman to send these messages.

For the control group, the village doctors provided their usual services.

### 2.4. Outcome Measures

Face-to-face questionnaire interviews were used to investigate the knowledge and use of folic acid supplements in both the control and intervention groups before and after the intervention. Compliance with folic acid supplement uses during the three months before pregnancy and in the first trimester were investigated as the primary outcome. “Compliance with folic acid use” was defined as using folic acid for more than 3 weeks in one month. The secondary outcome was the folic acid knowledge score, which was based on five indicators assessing whether the participants knew (1) that folic acid prevents NTDs; (2) that folic acid from food sources was not sufficient; (3) the best timing to start using folic acid; (4) the correct duration recommended by Chinese SFAPN for folic acid supplement use: 3 months before to 3 months after conception; and (5) the recommended daily dose of folic acid.

A score of 1 was assigned for correct answers and 0 for wrong answer or “don’t know”. The scores varied from 0–5 points and were classified into three levels: score 4 and above (cut-off 70%–100%); score 2–3 (cut-off 50%–70%); score 0–2 (less than 49%).

### 2.5. Statistical Analysis

Data cleaning and analysis were conducted using the SPSS 19.0 software package (SPSS Inc., Chicago, IL, USA). The awareness of and compliance with folic acid use were analyzed using Pearson’s chi-squared test and reported by the groups of women who were currently pregnant, gave birth in the last 12 months, or planned to have a baby. Logistic regression was used to examine the influence of various factors on whether the women continued to use folic acid.

## 3. Results

### 3.1. Sample Characteristics

A set of questionnaires was administered to the 759 participants of the target villages, and 757 completed questionnaires were returned for a response rate of 99.7%. Most of the women surveyed were of Han ethnicity (98.7%). The median age of the participants was 26 years, and the interquartile range was 24–30 years. Of the respondents, 43.7% were farmers, and 32.5% were employed. Approximately half of the women (45.6%) did not attain a high school diploma or post-high school degree. Approximately 19.4% of the participants reported a per capita annual net income below the Changsha county average level for 2012 (17,070 RMB). Among the 757 women planning pregnancies, 435 women (57.7%) had delivered their first child, 36.1% were planning their first pregnancy, and the others had had more than two previous live births. There were no significant differences in the women’s demographic characteristics between the intervention and control group.

### 3.2. Knowledge Regarding Folic Acid at Baseline

The baseline investigation showed that 742 out of 757 women (98.0%) had heard about folic acid (Table 1). Nearly 80% were aware that folic acid supplements were recommended before and during early conception, but only 32.8% knew that folic acid specifically helps to decrease the risk of NTDs. No differences were found between the control group and intervention group. Among the 742 women who reported “have heard about folic acid”, the main source of folic acid information was the village family planning staff (64.0%) or village doctors (52.0%).

### 3.3. Repeated Measures ANOVA of Knowledge Regarding Folic Acid after Intervention

At the final follow-up, 38 women in the control group and 70 women in the intervention group had dropped out. At baseline, there were no significant differences between the folic acid knowledge scores in the intervention and control groups (*t* = 0.547, *p* = 0.585). One year after intervention, knowledge scores increased in both intervention and control group. Mean knowledge scores increased by 18.9% (3.07 to 3.65) in the intervention group and by 7.7% (3.11 to 3.35) in the control group. As shown in Table 2, repeated measures ANOVA results indicated a significant interaction between intervention and time (*F* = 12.232, *p* = 0.001). Knowledge score increased faster in the intervention group than in the control group. This revealed that the intervention has an impact on increasing knowledge regarding folic acid over time.

### 3.4. Use of Folic Acid after Intervention

Before the intervention, 239 out of 338 women (70.7%) in the intervention group and 260 out of 357 women (72.8%) in the control group reported having received folic acid supplements, with no difference between the two groups. Similar compliance with folic acid supplement use (less than 30%) was observed for both groups before intervention. Folic acid education, follow-up, and monitoring of folic acid use and SMS messages containing folic acid information were provided for the intervention group for one year. As presented in Table 3, the majority of the women in the intervention group (85.4%) reported having obtained folic acid supplements after intervention, significantly higher than the control group (68.6%). Compliance with folic acid use was also significantly higher in the intervention group (41.7%–59.2%) compared with the control group (17.0%–29.4%).

### 3.5. Effects of the Intervention on Use of Folic Acid

At the final follow-up, 164 women self-reported having complied with the folic acid supplement recommendations for 6 months—three months before to three months after conception. We applied multivariable binary logistic regression models to identify the effects of the intervention and other factors on compliance with folic acid supplement recommendations. A total of 10 variables with a *p*-value ≤0.10 in the bivariate analysis were entered into the multivariable logistic regression analysis. As presented in Table 4, the women who participated in an intervention that integrated nutrition education, SMS information, and family planning staff visits were more likely to use folic acid supplements. We also found that women with family support and higher folic acid knowledge score were significantly more likely to use folic acid supplements.

## 4. Discussion

The present study found a wide deficiency in folic acid knowledge and the use of folic acid supplements. A large proportion of the sample was unaware of the need for folic acid supplementation. While most of the respondents indicated that they had heard of folic acid, only 32.8% knew that it could protect against NTDs. Similar results were found in other studies. In a survey of 2094 pregnant women in rural regions of western China, 56.0%–80.6% had heard about folic acid, but only 18.4%–38.9% indicated knowledge of the benefits of folic acid supplements [17]. In a study of 1907 rural women of childbearing age in Jiangsu province, approximately 99.6% of participants reported that they had heard of folic acid, but only 33.2%–43.8% knew that folic acid can prevent NTDs [18]. The use of folic acid supplements was also poor in this study; approximately 70% of the women had never taken folic acid supplements, and the compliance was less than 30%. Factors that contribute to this poor compliance may include inequalities in health resources, poor awareness and knowledge regarding folic acid, unplanned pregnancy, and other personal characteristics.

The best way to prevent NTDs is to ensure that rural women of childbearing age are taking appropriate amounts of folic acid daily. This requires them to be aware of when and where to obtain the folic acid supplements. Women in our study with higher folic acid knowledge scores were 2.7 times more likely to comply with folic acid recommendations, which was similar to findings of another study [19]. Strategies to increase the awareness of folic acid supplementation to prevent NTDs among rural women are important. In the SFAPN program, village doctors oversee nutrition education, providing folic acid supplements and following up on the use of folic acid. However, low education levels and insufficient professional training are barriers for delivering health information [20]. Some previous studies evaluated the effect of nutrition education on folic acid use among pregnant women, but only included pregnant women or doctors in town hospitals or county hospitals [21,22,23]. This study developed integrated interventions for both rural women and village health workers. Very few studies addressed folic acid training among Chinese village doctors. As we showed in another article, short-term training could effectively increase their knowledge regarding folic acid [24]. The training enhanced folic acid education in village clinics and promoted compliance with folic acid recommendations among rural women. SMS interventions have been shown to improve outcomes in rural health care settings [25]. Our study also indicated that SMS text messages encouraged rural women to use folic acid supplements (OR = 2.578, *p* = 0.006). In addition to providing reminders, the periodic SMS text messages sent information about folic acid to the rural women. Integrating SMS text messages into village-based health education may promote the delivery of folic acid information.

Village doctors are the gatekeepers of rural health care. However, gender imbalance is a barrier to women’s health care in some rural regions of China. Influenced by custom, some rural women will not seek maternal and gynecological care from a male health worker. In our study, only 23.3% of village doctors were female, similar to the findings of another study [26]. We hypothesized that among family planning staff, a female with a high reputation in the village may play an important role in the intervention. The family planning staff’s job includes regular monthly or bi-monthly follow-up visits to record the conditions of rural women’s pregnancies and improve management. It is easy for family planning staff to follow up and monitor folic acid use in their day-to-day work. Our results showed that women who received visits from family planning staff were 3.8 times more likely to comply with folic acid recommendations. Therefore, family planning staff should be considered the main implementers of SFAPN and other female health care interventions.

Our study has several strengths. To our knowledge, it is one of few that has developed an integrated folic acid intervention for both health care providers and health care recipients. We prepared detailed intervention plans. We worked with local health departments and developed a set of folic acid education materials that is still being used to train primary health workers. The communication service for delivering SMS text messages was open source and low cost.

The study also had some limitations inherent in its design. We only chose one site to implement the intervention. It was not powered to identify the effect of the intervention on the compliance with folic acid recommendations among women with low literacy. In addition, because we did not require responses to the SMS messages or reports of having taken the folic acid supplements, it was difficult to determine the exact effect of the SMS intervention. Furthermore, a one-year follow-up was too short to observe the effect of the intervention on birth defects.

## 5. Conclusions

The main barriers for SFAPN implementation include a lack of professional training, a lack of village doctors, time conflicts and work responsibilities, and gender imbalance. Our integrated intervention increased folic knowledge and compliance with folic acid supplement use among rural women. Future studies should be conducted to evaluate which components of the interventions are most effective. Additionally, further studies need to assess the effectiveness of the intervention for reducing birth defects.

## Figures and Tables

**Table 1:** Awareness and knowledge of folic acid among the rural women at baseline.

Knowledge Questions	Control Group (*n* = 384)	Intervention Group (*n* = 373)	Total (*n* = 757)
Have heard about folic acid	377 (98.2%)	365 (97.9%)	742 (98.0%)
Know that “Using of folic acid can prevent NTDs”	122 (31.7%)	126 (33.8%)	248 (32.8%)
Know that “Food sources cannot provide enough folic acid“	168 (43.8%)	151 (40.5%)	319 (42.1%)
Know “Best timing to start using folic acid”	302 (78.6%)	300 (80.4%)	602 (79.5%)
Know “the recommended duration of folic acid use (3 months before to 3 months after conception “	303 (78.9%)	308 (82.6%)	611 (80.8%)
Know the recommended daily dose of folic acid	330 (85.9%)	316 (84.7%)	646 (85.4%)
Source of folic acid information *			
Village family planning staff	243 (64.6%)	232 (63.4%)	475 (64.0%)
Village doctors	195 (51.9%)	191 (52.2%)	386 (52.0%)
Family/Friends	58 (15.4%)	60 (16.4%)	118 (15.9%)
Other	47 (12.5%)	43 (11.7%)	90 (12.1%)

* women who reported “have heard about folic acid”, *n* = 742 (376 in control group and 366 in intervention group).

**Table 2:** Repeated measures ANOVA for intervention and time for knowledge regarding folic acid.

Group	Before Intervention Mean ± SD	12-Months after Intervention Mean ± SD	Repeated Measures ANOVA		
			Between Subjects (Group)	Time Effect (Within Group Comparison)	Time × Intervention Effect (Between Group Comparison)
Control (*n* = 347)	3.11 ± 1.15	3.35 ± 0.99	*F* = 3.293 *p* = 0.070	*F* = 53.966 *p*< 0.01	*F* = 12.232 *p* = 0.001
Intervention (*n* = 302)	3.07 ± 1.16	3.65 ± 1.00			

**Table 3:** Comparison of the intervention group with the control group regarding folic acid use among rural women after intervention.

Folic Acid Use	Control Group	Intervention Group	Chi-Square	*p*
	194 (68.6%)	239 (85.4%)	22.38	<0.01
Complied with folic acid supplement recommendations during the three months before pregnancy	57 (29.4%)	141 (59.2%)	38.39	<0.01
Complied with folic acid supplement recommendations during the first trimester	53 (27.7%)	133 (56.3%)	35.14	<0.01
Complied with folic acid supplement recommendations from 3 months before to 3 months after conception	32 (17.0%)	93 (41.7%)	29.36	<0.01

**Table 4:** Factors associated with compliance with folic acid supplement use from 3 months before to 3 months after conception among rural women according to multivariate logistic regression.

Variable	Women Who Complied with Folic Acid Supplement Recommendations 3 Months before to 3 Months after Conception						
	B	S.E.	Wald	*df*	Sig	OR	95% CI
Constant	−3.885	0.337	132.915	1	<0.001	0.021	
Received FA education at village clinics	1.246	0.261	22.703	1	<0.001	3.475	2.082–5.802
Received SMS intervention	0.947	0.344	7.568	1	0.006	2.578	1.313–5.063
Family planning staff followed-up and monitored FA use	1.339	0.179	55.923	1	<0.001	3.813	2.685–5.416
Family support	0.812	0.266	9.319	1	0.002	2.252	1.337–3.792
FA score above 4	0.840	0.236	17.580	1	<0.001	2.692	1.694–4.277

S.E. = standard error.

## Abbreviations

FA	Folic Acid
NTD	Neural Tube Defect
SFAPN	Supplementing Folic Acid to Prevent NTD
